# The New TNM Staging System for Thyroid Cancer and the Risk of Disease Downstaging

**DOI:** 10.3389/fendo.2018.00541

**Published:** 2018-09-18

**Authors:** Claudio Casella, Silvia Ministrini, Alessandro Galani, Francesco Mastriale, Carlo Cappelli, Nazario Portolani

**Affiliations:** ^1^Department of Molecular and Translational Medicine, University of Brescia, Brescia, Italy; ^2^Department of Clinical and Experimental Sciences, University of Brescia, Brescia, Italy

**Keywords:** thyroid cancer, staging system, downstaging, tumor invasion, TNM 2018

## Abstract

In October 2016 the American Joint Committee on Cancer (AJCC) published the 8th edition of the AJCC/TNM cancer staging system and it has been introduced in clinical practice since 1st January 2018. The effect of most of the changes in the new edition was the downstaging of a significant number of patients into lower stages, reflecting their low risk of thyroid cancer-related death. One of the most relevant modification refers to the role of the microscopic extra-thyroidal tumor invasion, which is no longer considered as criterion for the classification of T3 tumors. With the present study we want to assess the impact of the changes of the new staging system and therefore we analyzed or casistic of 84 T1-T3 thyroid-cancer patients. The results of our analysis show that he downstaging of patients according to the 8th TNM edition does not necessarily reflect less aggressive disease: we actually reported 2 lymph-nodal recurrences (40%) in the five patients that were downstaged from pT3 to pT2 and the lypmh-nodal recurrence rate for stage I rises from 0% with the 7th TNM edition to 5.3% with the 8th edition.

## Introduction

Thyroid cancer is the most common type of malignant endocrine cancer and its incidence is rising worldwide (1, 2). Differentiated thyroid cancer, which includes papillary and follicular cancers, comprises majority (90%) of all thyroid cancers (1, 3) and it has a favorable prognosis. However, a minority of patients develops locoregional recurrence, including cervical lymph node metastases (3).

Despite the increasing incidence of differentiated thyroid cancer, no change in mortality rate has been observed and this could be due to the fact that increased diagnosis of small tumors might not influence prognosis (4). The huge increase in the incidence of small and early-stage tumors is leading toward a less aggressive therapeutic approach, as reflected by the recommendations in the 2015 American Thyroid Association (ATA) guidelines (3). The American Joint Committee on Cancer/Union for International Cancer Control (AJCC/UICC) updated in 2017 the tumor node metastasis (TNM) staging system and came up with its 8th edition (5), introducing significant changes. The main changes include a rise in the age cutoff from 45 to 55 years and the removal of lymph node metastases from the T3 definition; moreover microscopic extrathyroidal extension is no longer considered for staging. As a result, a large proportion of patients are shifted to stage I.

With the present study we would like to analyze the impact of the new TNM classification on our thyroid cancer population of patients.

## Materials and methods

We retrospectively reviewed clinical records of 84 patients operated at our institution for T1-T3 thyroid cancer from September 2010 to December 2015. In all cases we performed total thyroidectomy without prophylactic central neck dissection as all patients presented no lymph nodal involvement at preoperative staging (3, 6–11).

Data were collected anonymously and with patients consent.

Radioactive iodine therapy was administered to 77.1% patients. Median age was 57.6 ± 13.4 years. The characteristics of the population and of the tumor are described in detail in Table 1, while the details about histopathological examination according to the 7 and the 8th Edition of TNM staging system (5, 12) are listed in Table 2.

**Table 1:** Patients characteristics.

	**Nr**.	**%**
**Total patients**	84	
Male	17	20.2
Female	67	79.8
Median age	57.6 ± 13.4 years	
Mean diameter	2.2 cm	
**THYROID FUNCTION**		
Euthyroidism	78	92.9
Hypothyroidism	5	5.9
Hyperthyroidism	1	1.2
**HISTOLOGY**		
Papillary	33	39.3
Follicular variant	45	53.6
Mixed papillary	6	7.1
Multifocal	40	47.6
Bilateral	14	16.7

**Table 2:** Tumor characteristics according to the 7 and 8th edition of TNM and data about recurrence.

	**7th TNM classification**		**8th TNM classification**		

	**Nr**.	**%**	**Nr**.	**%**	***p*****-value**
pT1	55	65.5	55	65.5	1
pT2	15	17.8	20	23.8	0.448
pT3	14	16.7	9	10.7	0.370
Stage I	55	65.5	75	89.3	<**0.001**
Stage II	15	17.8	9	10.4	0.270
Stage III	14	16.7	0	0	<**0.001**
**CERVICAL LYMPH NODE RECURRENCE AT FOLLOW-UP**					
Stage I	0/55	0	4/75	5.3	0.137
Stage II	2/15	13.3	0/9	0	0.511
Stage III	2/14	14.3	0/0	0	–

All patients were staged based on the AJCC TNM seventh edition system and compared to the recently published eighth edition. For the prediction of recurrence and/or persistence, patients were classified according to the 2015 modified ATA initial risk-stratification system (13).

Patients were monitored each 6 months by clinical examination, thyroid function tests, neck ultrasound, and serum Thyroglobulin dosage. Persistent or recurrent disease was diagnosed based on histopathology, imaging studies, and Thyroglobulin levels.

At follow-up (duration range: 27–80 months; mean: 67 months) we registered 4 cases (4,8%) of cervical lymph node recurrence after 9, 16, 18, and 32 months respectively; mortality rate was 0%. The recurrence was treated by radioactive iodine therapy.

### Statistical analysis

Categorical data are expressed as percentages while continuous variables are reported as mean and standard deviation. Chi-square and Fisher's Exact tests were used to evaluate the relationship between clinical characteristics. Statistical analysis is performed by means of SPSS® program.

## Results

According to the 8th edition of TNM staging system (5), 5 out of 14 (35,7%) patients do not meet the criteria for inclusion in the new pT3 definition, as they presented only microscopic extra-capsular invasion. These patients are therefore reclassified as pT2. Thanks to this change, our study population is now composed of 20 pT2 patients (23.8 vs. 17.8%) and 9 pT3 patients (10.7 vs. 16.7%). As regard the stage stratification, 55- 15- and 14 patients were classified in stage I, II and III of 7th TNM, respectively; according to 8th TNM, 75 patients are considered in stage I (89,3% vs. 65,5%) and nine patients in stage II (10.4 vs. 17.8%). Twenty patients (23.8%) are down-staged from stage II-III to stage I and nine patients (10.7%) from stage III to stage II (Table 2). According to ATA 2015 risk stratification system, the microscopic extrathyroidal extension amounts to an intermediate risk, so when comparing the 7 and 8th editions for stages I–II only, the proportion of intermediate-risk patients increased from 0 to 5.9% (0/70 vs. 5/84, *p* = 0.064).

Two out of five patients (40%) that were restaged from stage III to I, because they presented only minimal extra-thyroidal invasion, developed tumor recurrence in the central lymph node compartment; the rate of tumor recurrence is therefore 0 vs. 5.3% (0/55 vs. 4/75). The two patients that presented lymph node recurrence among the downstaged group developed the recurrence earlier than the two patients in the non-downstaged group (9 and 16 months vs. 18 and 32 months).

## Discussion

In October 2016 the American Joint Committee on Cancer (AJCC) published the 8th edition of the AJCC/Tumor-Lymph Node-Metastasis (TNM) cancer staging system (5), replacing the seventh edition, which has been in use since 2009 (12). Appropriate staging according to the 8th edition requires integration of a wide variety of information based on patient history, physical examination, imaging, intraoperative findings and pathologic data (14, 15).

The purpose of current study is to compare the 7 and 8th editions of the TNM staging system of thyroid cancer in our records of thyroid cancer patients.

A notable change in 8th TNM classification is the definition of the T3 thyroid cancer (5): according to TNM-8, T3 is defined as the presence of macroscopic extra-thyroidal tumor extension invading strep muscle. Such extra-thyroidal invasion is observed in 5–45% of patients and has been recognized as an important prognostic factor (16–19). Some studies had shown that massive extra-thyroidal invasion is associated with significantly worse survival (17, 20), while the presence of microscopic minimal extra-thyroidal extension did not seem to impact upon prognosis (21–26).

Therefore in the new TNM staging system, the microscopic extra-thyroidal invasion was down-staged, with the effort to reduce the overstaging and consequently the overtreatment of the disease.

In the current study, with the new TNM edition nearly one-third (34.5%) of patients were down-staged due to changes in T stage. The downgrading of microscopic extra-thyroidal invasion resulted in a shift of T3 patients from stage III to stage II and the proportion of intermediate risk patients (according to ATA-2015 classification) in stages I–II increased, resulting in 6% more patients at higher risk within this stage category.

Also Kim et al. (27) found out in their study on 1613 patients that, when 8th TNM version was applied, 38% of patients were reclassified into lower TNM stages and 63% of patients with T3 classification were restaged as T1 or T2. The disease specific survival of patients in stages III and IV according to TNM-8 was worse than those according to TNM-7 (98.8 and 83.2% for TNM-7 vs. 72.3 and 48.6% for TNM-8, respectively) and they concluded that TNM-8 had better predictability for disease specific survival compared to TNM-7 because the new staging system could reduce the number of patients in stages III and IV. Suh et al. (1) compared different staging systems for thyroid cancer and they concluded that the 8th edition ofAJCC was the most accurate in predicting patient outcome.

In the study from Shteinshnaider et al. (28) on a wide population, the proportion of intermediate/high risk patients in stages I–II according to the 8th edition increased considerably compared to the previous edition. Patients reclassified according to the 8th edition in the stage II had more lymph node metastases, more intermediate and high recurrence risk, more reoperations, more persistency of disease and non-significant increase in disease specific mortality compared to the previous edition. These results are in line with our observation.

### Limitations and further perspective

We are well aware that our records are limited and their statistical power is low. We expect however that this paper could represent an encouragement for further studies on a wider population in order to corroborate our findings.

## Conclusion

Although we recognize the low statistical power of the current study, the results of our analysis show that the downstaging of patients according to the 8th TNM edition does not necessarily reflect less aggressive disease: we actually reported 2 lymph-nodal recurrences (40%) in the five patients that were downstaged from pT3 to pT2 and the lypmh-nodal recurrence rate for stage I rose from 0% with the 7th TNM edition to 5.3% with the 8th edition. These patients should be approached carefully and at least a close follow-up should be performed in the cases in whom the new staging system entails a downstaging from pT3 to pT2 due to microscopic extrathyroidal invasion.

However we advocate the necessity of studies on more numerous population of patients in order to validate our preliminary results and reinforce the strength of our observations. Moreover we suggest that also the role of radioiodine therapy could be investigated in patients at low risk of relapse (29).

## Data availability

The raw data supporting the conclusions of this manuscript will be made available by the authors, without undue reservation, to any qualified researcher.

## Ethics statement

According to current Italian Legislation the Approval of the Ethics Commitee for a retrospective observational study is not required.

## Author contributions

CLC and CAC contributed to the conception and design of the work. CLC and SM participated to data analysis and text editing. FM and AG participated to data collection and patients' follow-up. CLC and NP contributed to text revision.

### Conflict of interest statement

The authors declare that the research was conducted in the absence of any commercial or financial relationships that could be construed as a potential conflict of interest.

## References

[1] Suh S Kim YH Goh TS Lee J Jeong DC Oh SO. Outcome prediction with the revised American joint committee on cancer staging system and American thyroid association guidelines for thyroid cancer. Endocrine (2017) 58:495–502. 10.1007/s12020-017-1449-4 29030773

[2] Cabanillas ME McFadden DG Durante C. Thyroid cancer. Lancet (2016) 388:2783–95 10.1016/S0140-6736(16)30172-6 27240885

[3] Haugen BR Alexander EK Bible KC Doherty GM Mandel SJ Nikiforov YE. 2015 American Thyroid Association management guidelines for adult patients with thyroid nodules and differentiated thyroid cancer. The American Thyroid Association Guidelines task force on thyroid nodules and differentiated thyroid cancer. Thyroid (2016) 26:1–133. 10.1089/thy.2015.0020 26462967 PMC4739132

[4] Davies L Welch HG. Increasing incidence of thyroid cancer in the United States, 1973–2002. JAMA (2006) 295:2164–7. 10.1001/jama.295.18.2164 16684987

[5] Amin MB Edge S Greene F Byrd DR Brookland RK Washington MK. AJCC Cancer Staging Manual. 8th edn. New York, NY: Springer (2017).

[6] Conzo G Tartaglia E Avenia N Calò PG de Bellis A Esposito K. Role of prophylactic central compartment lymph node dissection in clinically N0 differentiated thyroid cancer patients: analysis of risk factors and review of modern trends. World J Surg Oncol. (2016) 14:149. 10.1186/s12957-016-0879-4 27185169 PMC4869299

[7] Mitchell AL Gandhi A Scott-Coombes D Perros P. Management of thyroid cancer: United Kingdom National Multidisciplinary Guidelines. J Laryngol Otol. (2016) 130(Suppl. S2), S150–60. 10.1017/S0022215116000578 27841128 PMC4873931

[8] Yuan J Li J Chen X Lin X Du J Zhao G. Identification of risk factors of central lymph node metastasis and evaluation of the effect of prophylactic central neck dissection on migration of staging and risk stratification in patients with clinically node-negative papillary thyroid microcarcinoma. Bull du Cancer (2017) 104:516–23. 10.1016/j.bulcan.2017.03.005 28476312

[9] Yoo HS Shin MC Ji YB Song CM Lee SH Tae K. Optimal extent of prophylactic central neck dissection for papillary thyroid carcinoma: comparison of unilateral versus bilateral central neck dissection. Asian J Surg. (2017) 41:363–9. 10.1016/j.asjsur.2017.03.002 28454667

[10] Gui CY Qiu SL Peng ZH Wang M. Clinical and pathologic predictors of central lymph node metastasis in papillary thyroid microcarcinoma: a retrospective cohort study. J Endocrinol Invest. (2018) 41:403–9. 10.1007/s40618-017-0759-y 28884301

[11] Nixon IJ Wang LY Ganly I Patel SG Morris LG Migliacci JC. Outcomes for patients with papillary thyroid cancer who do not undergo prophylactic central neck dissection. Br J Surg. (2016) 103:218–25. 10.1002/bjs.10036 26511531 PMC4976488

[12] Edge SB Compton CC. The american joint committee on cancer: the 7th edition of the AJCC cancer staging manual and the future of TNM. Ann Surg Oncol. (2010) 17:1471–4. 10.1245/s10434-010-0985-4 20180029

[13] Nixon IJ Wang LY Migliacci JC Eskander A Campbell MJ Aniss A. An International multi-institutional validation of age 55 years as a cutoff for risk stratification in the AJCC/UICC staging system for well-differentiated thyroid cancer. Thyroid (2016) 26:373–80. 10.1089/thy.2015.0315 26914539 PMC4790212

[14] Perrier ND Brierley JD Tuttle RM. Differentiated and anaplastic thyroid carcinoma: major changes in the American Joint Committee on Cancer eighth edition cancer staging manual. CA Cancer J Clin. (2018) 68:55–63. 10.3322/caac.21439 29092098 PMC5766386

[15] Durante C Grani G Lamartina L Filetti S Mandel SJ Cooper DS. The diagnosis and management of thyroid nodules: a review. JAMA (2018) 319:914–24. 10.1001/jama.2018.0898 29509871

[16] Ortiz S Rodriguez JM Soria T Perez-Flores D Pinero A Moreno J. Extrathyroid spread in papillary carcinoma of the thyroid: clinicopathological and prognostic study. Otolaryngol Head Neck Surg. (2001) 124:261–5. 10.1067/mhn.2001.113141 11240987

[17] Shaha AR. Implications of prognostic factors and risk groups in the management of differentiated thyroid cancer. Laryngoscope (2004) 114:393–402. 10.1097/00005537-200403000-00001 15091208

[18] Ito Y Tomoda C Uruno T Takamura Y Miya A Kobayashi K. Minimal extrathyroid extension does not affect the relapse-free survival of patients with papillary thyroid carcinoma measuring 4 cm or less over the age of 45 years. Surg Today (2006) 36:12–18. 10.1007/s00595-005-3090-8 16378187

[19] Andersen PE Kinsella J Loree TR Shaha AR Shah JP. Differentiated carcinoma of the thyroid with extrathyroidal extension. Am J Surg. (1995) 170:467–70. 10.1016/S0002-9610(99)80331-6 7485734

[20] Ito Y Tomoda C Uruno T Takamura Y Miya A Kobayashi K. Prognostic significance of extrathyroid extension of papillary thyroid carcinoma: massive but not minimal extension affects the relapse-free survival. World J Surg. (2006) 30:780–6. 10.1007/s00268-005-0270-z 16411013

[21] Woo CG Sung CO Choi YM Kim WG Kim TY Shong YK. Clinicopathological significance of minimal extrathyroid extension in solitary papillary thyroid carcinomas. Ann Surg Oncol. (2015) 22:S728–33. 10.1245/s10434-015-4659-0 26077913 PMC4686556

[22] Nixon IJ Ganly I Patel S Palmer FL Whitcher MM Tuttle RM. The impact of microscopic extrathyroid extension on outcome in patients with clinical T1 and T2 well-differentiated thyroid cancer. Surgery (2011) 150:1242–9. 10.1016/j.surg.2011.09.007 22136847 PMC4151609

[23] Yin DT Yu K Lu RQ Li X Xu J Lei M. Prognostic impact of minimal extrathyroidal extension in papillary thyroid carcinoma. Medicine (2016) 95:e5794. 10.1097/MD.0000000000005794 28033304 PMC5207600

[24] Lang BH Shek TW Wan KY. Does microscopically involved margin increase disease recurrence after curative surgery in papillary thyroid carcinoma? J Surg Oncol. (2016) 113:635-9. 10.1002/jso.24194 26843438

[25] Castagna MG Forleo R Maino F Fralassi N Barbato F Palmitesta P. Small papillary thyroid carcinoma with minimal extrathyroidal extension should be managed as ATA low-risk tumor. J Endocrinol Invest. (2018) 41:1029–35. 10.1007/s40618-018-0854-8 29470826

[26] Lamartina L Grani G Arvat E Nervo A Zatelli MC Rossi R. 8th edition of the AJCC/TNM staging system of thyroid cancer: what to expect (ITCO#2). Endocr Relat Cancer. (2018) 25:L7–11. 10.1530/ERC-17-0453 29192093

[27] Kim M Kim WG Oh HS Park S Kwon H Song DE. Comparison of the seventh and eighth editions of the american joint committee on cancer/union for international cancer control tumor-node-metastasis staging system for differentiated thyroid cancer. Thyroid (2017) 27:1149–55. 10.1089/thy.2017.0050 28635571

[28] Shteinshnaider M Muallem Kalmovich L Koren S Or K Cantrell D Benbassat C. Reassessment of differentiated thyroid cancer patients using the eighth TNM/AJCC classification system: a comparative study. Thyroid (2018) 28:201–9. 10.1089/thy.2017.0265 29256827

[29] Schlumberger M Leboulleux S Catargi B Deandreis D Zerdoud S Bardet S. Outcome after ablation in patients with low-risk thyroid cancer (ESTIMABL1): 5-year follow-up results of a randomised, phase 3, equivalence trial. Lancet Diabetes Endocrinol. (2018) 6:618–26. 10.1016/S2213-8587(18)30113-X 29807824

